# Evidence for and localization of proposed causative variants in cattle and pig genomes

**DOI:** 10.1186/s12711-021-00662-x

**Published:** 2021-08-30

**Authors:** Martin Johnsson, Melissa K. Jungnickel

**Affiliations:** 1 Department of Animal Breeding and Genetics, Swedish University of Agricultural Sciences, Box 7023, 750 07 Uppsala, Sweden; 2 The Roslin Institute and Royal (Dick) School of Veterinary Studies, The University of Edinburgh, Midlothian, EH25 9RG Scotland, UK

## Abstract

**Background:**

This paper reviews the localization of published potential causative variants in contemporary pig and cattle reference genomes, and the evidence for their causality. In spite of the difficulties inherent to the identification of causative variants from genetic mapping and genome-wide association studies, researchers in animal genetics have proposed putative causative variants for several traits relevant to livestock breeding.

**Results:**

For this review, we read the literature that supports potential causative variants in 13 genes (*ABCG2*, *DGAT1*, *GHR*, *IGF2*, *MC4R*, *MSTN*, *NR6A1*, *PHGK1*, *PRKAG3*, *PLRL*, *RYR1*, *SYNGR2* and *VRTN*) in cattle and pigs, and localized them in contemporary reference genomes. We review the evidence for their causality, by aiming to separate the evidence for the locus, the proposed causative gene and the proposed causative variant, and report the bioinformatic searches and tactics needed to localize the sequence variants in the cattle or pig genome.

**Conclusions:**

Taken together, there is usually good evidence for the association at the locus level, some evidence for a specific causative gene at eight of the loci, and some experimental evidence for a specific causative variant at six of the loci. We recommend that researchers who report new potential causative variants use referenced coordinate systems, show local sequence context, and submit variants to repositories.

## Background

Identification of causative variants from genetic mapping and genome-wide association studies is a difficult problem, especially for quantitative traits. The difficulties stem from several unfortunate facts of genetics. Quantitative traits are affected by many variants each with a small effect, which limits the power of genetic mapping, even with large sample sizes. The genomic resolution of genetic mapping is also limited by the correlation between genetic variants (linkage disequilibrium), meaning that there are many candidate genes and variants for each association. Especially in commercial livestock breeds that have seen systematic breeding, familial relationship leads to linkage disequilibrium that can extend over 100 kb (reviewed by [1]). Furthermore, while genetic mapping studies follow relatively standardized linkage mapping or genome-wide association workflows, there is no clear recipe for the experimental biology studies that are needed to go from the associated locus to the causative variant.

In spite of these difficulties, researchers in animal genetics have isolated a small number, probably less than 50, putative causative variants for traits relevant to livestock breeding (reviewed for example by [1–3]). The variants and the evidence that support them are documented in a somewhat ad hoc fashion in scientific papers and databases.

As larger datasets of genotyped and sequenced livestock animals that are phenotyped for complex traits, as well as functional genomic data from livestock, are accruing [4], we might expect a new boom in the identification of causative variants. Large datasets increase the power to detect loci for quantitative traits, and sequence data will allow them to be fine-mapped down to the limit set by linkage disequilibrium. New functional genomic assays will make it easier to take candidate variants identified from fine-mapping studies further and to test their effects in the laboratory. In particular, more comprehensive open chromatin data (e.g. ATAC-seq or ChIP-seq of histone marks) from multiple tissues (e.g. [5–7]), and expression quantitative trait locus studies (e.g. [8–11]) will help prioritize non-coding variants, that are likely to explain a substantial part of the genetic variation in quantitative traits (quantified by [12] in cattle), but with effects that are challenging to predict from DNA sequence. We expect that this will make challenges in the identification of causative variants more pressing in the near future.

For the purpose of this paper, a “locus” refers to a region of a genome associated with a trait, a “causative variant” refers to a sequence variant that causes such a genetic association, and a “causative gene” is the gene that mediates that causative effect. In the literature, loci are sometimes specified as “quantitative trait loci” and causative variants are variously referred to as “quantitative trait nucleotides (QTN)”, “causative mutations” or “causal variants”. For our purposes, these terms are interchangeable.

For this review, we read the literature that supports potential causative variants in 13 genes (*ABCG2*, *DGAT1*, *GHR*, *IGF2*, *MC4R*, *MSTN*, *NR6A1*, *PHGK1*, *PRKAG3*, *PLRL*, *RYR1*, *SYNGR2* and *VRTN*) in cattle and pigs, and localized them in contemporary reference genomes. Most of them are single nucleotide variants (SNVs), and some are short insertions/deletions (indels). We have concentrated on causative variants that have been proposed for economically important traits, in particular for quantitative traits, but also included a few major genetic defects; however, we have excluded causative variants for breed-type traits such as pigmentation, and recessive lethal haplotypes. We review the evidence for causality, aiming to separate the evidence for the locus, for the proposed causative gene and for the proposed causative variant, and report the bioinformatic searches and tactics needed to localize the sequence variants in the cattle or pig genome. We hope that this paper will be useful to researchers confronted with the task of following up on established genetic mapping results, and point out what information might be helpful to include when reporting new candidate causative variants.

## Main text

To localise putative causative variants in contemporary reference genomes, we used the Ensembl Genes [13] and Ensembl Variation [14] database version 102. The livestock genomics resources provided by Ensembl and how to use them have recently been reviewed by Martin et al. [15]. The Ensembl Variation database contains SNVs that are imported from the dbSNP database that have been remapped to the reference genome in question, and have had consequences predicted with the Ensembl Variant Effect Predictor [16]; in one case, we generated predictions by inputting a modified sequence into the VEP web interface. However, the dbSNP database has discontinued non-human animals, and has been superseded by the European Variation Archive (https://www.ebi.ac.uk/eva/) as a repository for livestock genetic variants. For a few of the variants, which can be considered as responsible for genetic disorders or monogenic traits, there are entries in the Online Mendelian Inheritance In Animals (OMIA) database [17] (https://www.omia.org), which are also listed in Tables 1 and 2. The reference genome versions used were ARS-UCD1.2 for cattle [18] and Sscrofa11.1 for pig [19]. In one case where the gene was missing from the Ensembl gene annotation, we used the NCBI gene annotation instead (https://www.ncbi.nlm.nih.gov/genome/annotation_euk/process/) [20].

**Table 1:** Proposed causative variants in cattle

Gene (variants)	Trait (species)	Variant type	Variant codes	Effect of alleles	Position (ARS-UCD1.2)	Variant Acc	OMIA Acc	Original citation
*ABCG2* (p.Tyr581Ser)	Milk yield and composition	Missense SNV	*NC_037333.1:g.36599640A* > *C* ENSBTAP00000051068:p.Tyr581Ser	*A* has higher milk yield and lower fat and protein concentration	6:36,599,640			[24]
*DGAT1* (p.Ala323Lys)	Milk yield and composition	Non-synonymous multinucleotide variant	*NC_037341.1:g.611019_611020delinsAA* ENSBTAP00000037256:p.Ala323Lys	*AA* has higher milk yield and lower fat and protein concentration	14:611,019–611,020			[37]
*GHR* (p.Phe279Tyr)	Milk yield and composition	Missense SNV	*NC_037347.1:g.31870098 T* > *A* ENSBTAP00000001758:p.Phe279Tyr	*T* has higher milk yield and lower fat and protein concentration	20:31,870,098	rs385640152		[45]
*MSTN* (p.Asp273fs)	Muscle growth (cattle)	Frameshift deletion	*NC_037329.1:g.6283674_6283684del* ENSBTAP00000015674:p.Asp273fs	Deletion has more muscle	2:6,283,674–6,283,684	rs382669990	OMIA 000,683–9913	[48–50]
(p.Gln204*)		Stop gain SNV	*NC_037329.1:g.6281434C* > *T* ENSBTAP00000015674:p.Gln204*	*T* has more muscle	2:6,281,434	rs110344317		
(p.Glu226*)		Stop gain SNV	*NC_037329.1:g.6281500G* > *T* ENSBTAP00000015674:p.Glu226*	*T* has more muscle	2:6,281,500			
(p.Cys313Tyr)		Missense SNV	*NC_037329.1:g.6283794G* > *A* ENSBTAP00000015674:p.Cys313Tyr	*A* has more muscle	2:6,283,794			
(p.Phe140*)		Stop gain indel	*NC_037329.1:g.6281243_6281249delinsAAGCATACAA* ENSBTAP00000015674:p.Phe140*	The long allele has more muscle	2:6,281,243–6,281,249			
*PRLR* (p.Ala461fs)	Slick coat type	Frameshift deletion	*NC_037347.1:g.39099214_delC* ENSBTAP00000069979:p.Ala461fs	Deletion has slick coat	20:39,099,214	rs517047387	OMIA 001,372–9913	[64, 65]
(p.Ser465*)		Stop gain SNV	*NC_037347.1:g.39099226C* > *A* ENSBTAP00000069979:p.Ser465*	*A* has slick coat	20:39,099,226			
(p.Arg497*)		Stop gain SNV	*NC_037347.1:g.39099321C* > *T* ENSBTAP00000069979:p.Arg497*	*T* has slick coat	20:39,099,321			

When there are multiple transcripts associated with a nonsynonymous variant that all give rise to the same coding consequences, we give a variant code only relative to one of them. When the gene is not available in the Ensembl gene annotation, we use NCBI annotation instead. We give variant accessions (Acc) (from dbSNP, now available in Ensembl Variation and EVA), and Online Mendelian Inheritance in Animals (OMIA) accessions (Acc) where available

**Table 2:** Proposed causative variants in pigs

Gene (variants)	Trait (species)	Variant type	Variant codes	Effect of alleles	Position (Sscrofa11.1)	Variant Acc	OMIA Acc	Original citation
*IGF2* (*g.1483817T* > *C*)	Muscle growth	Intronic SNV	*NC_010444.4:g.1483817 T* > *C*	*T* has higher growth and lean meat production	2:1,483,817			[74]
*MC4R* (p.Asp298Asn)	Growth and fatness	Missense SNV	*NC_010443.5:g.160773437G* > *A* ENSSSCP00000074588:p.Asp298Asn	*A* has faster growth and more fat	1:160,773,437	rs81219178		[82]
*MSTN* (p.Glu274*)	Leg weakness	Stop gain SNV	*NC_010457.5:g.94623834C* > *A* ENSSSCP00000017001:p.Glu274*	*A* has leg weakness	15:94,623,834		OMIA 002,161–9823	
*NR6A1* (p.Leu192Pro)	Vertebrae number	Missense SNV	*NC_010443.5:g.265347265A* > *G* ENSSSCP00000005986:p.Leu192Pro	*A* has higher vertebrate number	1:265,347,265	rs326780270		[97]
*PHKG1 (g.16830320C* > *A)*	Glycogen content and meat quality	Splice region SNV	*NC_010445.4:g.16830320C* > *A*	*A* has higher glycogen content	3:16,830,320	rs330928088		[103]
*PRKAG3* (p.Arg250Gln)	Glycogen content and meat quality	Missense SNV	*NC_010457.5:g.120863533C* > *T* ENSSSCP00000017163:p.Arg250Gln	*T* has high glycogen content	15:120,863,533	rs1109104772	OMIA 001,085–9823	[104]
*RYR1* (p.Arg651Cys)	Malignant hypothermia	Missense SNV	*NC_010448.4:g.47357966 T* > *C* NP_001001534.1:p.Arg651Cys	*T* is halothane sensitive	6:47,357,966	rs344435545	OMIA 000,621–9823	[114, 117]
*SYNGR2* (p.Cys63Arg)	Porcine circovirus viral load	Missense SNV	*NC_010454.4:g3797515A* > *C* ENSSSCP00000041695:p.Cys63Arg	*A* has lower viral load	12:3,797,515			[118]
*VRTN (g.97614602A* > *C)*	Vertebrae number	Noncoding SNV	*NC_010449.5:g.97614602A* > *C*	*C* has more vertebrae	7:97,614,602	rs709317845		[102]
*(g.97615879_97615880ins)*		Noncoding indel	*NC_010449.5:g.97615879_97615880ins[AB554652.1:20312_20602]*	Insertion has more vertebrae	7:97,615,880			

When there are multiple transcripts associated with a nonsynonymous variant that all give rise to the same coding consequences, we give a variant code only relative to one of them. When the gene is not available in the Ensembl gene annotation, we use NCBI annotation instead. We give variant accessions (from dbSNP, now available in Ensembl Variation and EVA), and Online Mendelian Inheritance in Animals (OMIA) accessions where available

Using the Ensembl genome browser, we looked for variants in the gene that matched the original description (position on the protein, and amino acid substitution) in any of the Ensembl transcripts associated with the gene. We looked in Ensembl Variation for variants with literature citations. When the original publications gave the sequence (amino acid or nucleotide) close to the variant, we verified the position by pairwise alignment of amino acid sequences with the Emboss program Needle for global alignment or the Emboss program Water for local alignment [21], or by alignment of nucleotide sequences to the genome with the BLAT program [22]. We used the Ensembl REST API web service to map coordinates of the amino acid positions in the Ensembl gene database to the reference genomes [23]. We used the LiftOver tool of the UCSC genome browser to map coordinates between reference genome versions when coordinates were given for an older reference genome (https://genome.ucsc.edu/cgi-bin/hgLiftOver).

### Proposed causative variants

Below, we report the localisation and citation for each of the potential causative variants, and comment on the evidence that supports these variants. Tables 1 and 2 list the variants and their localisation in the cattle and pig genome, respectively. We will discuss the evidence for each variant at three levels:

- whether the proposed gene is the causative gene mediating the genetic effect at the locus;
- whether the specific variant proposed is the causative variant;
- and, whether the locus has been replicated in genome scans, i.e., whether associations to similar traits have been detected in later genome-wide studies.

#### Cattle

##### *ABCG2* (ENSBTAP00000051068:p.Tyr581Ser)

The p.Tyr581Ser missense variant in the *ATP-binding cassette transporter G2* (*ABCG2*) gene is a putative causative variant involved in milk yield and composition in cattle. It was first identified by [24] and has been associated with the secretion of enterolactone, riboflavin and uric acid in milk [25]. ABCG2 is a transport protein involved in the secretion of several drugs in human milk [26, 27].

The *ABCG2* locus was the subject of debate (see commentary in [28]) because another candidate gene *osteopontin* was reported at this locus [29], with an insertion/deletion (indel) that was concordant with the locus. However, a later study refined this locus, which excluded *osteopontin* [30].

*Location* The p.Tyr581Ser variant is located on cattle chromosome 6 at position 36,599,640 in ARS-UCD1.2. The variant was mapped to the genome by mapping amino acid position 581 of the peptide sequence ENSBTAP00000051068 to the genome sequence with the Ensembl REST API. This gives the position of the codon that matches this amino acid (6:36,599,639–36,599,641). The middle bp of the codon is an A that gives rise to a Tyr > Ser substitution when changed to a C. Thus, it matches the A > C substitution in the original publication. We verified the localization by aligning the amino acid sequence in Fig. 4 of Cohen-Zinder et al. [24] to the Ensembl peptide ENSBTAP00000051068 with Emboss Needle.

*Evidence for the causative gene* Given the known function of the product of this gene in the secretion of small molecules in milk, it is biologically plausible. Cohen-Zinder et al. [24] measured the expression of genes within the candidate region in the mammary gland during lactation and during the dry period, and identified three genes—*SPP1*, *PKD2* and *ABCG2*—that were differentially expressed. The authors demonstrated that expression of *SSPP1* and *ABCG2* in the bovine mammary gland increased from parturition through lactation and used this data as evidence that the bovine *ABCG2* and *SSP1* genes play a role in the mammary gland during lactation.

*Evidence for the causative variant* Cohen-Zinder et al. [24] sequenced the coding regions of these three genes, and found one variant that was concordant with an effect on milk fat and protein composition (N = 341 sires). They aligned the amino acid sequence with that of homologous genes from other species. They identified a substitution that occurred in an apparently conserved domain, and that introduced an amino acid with different chemical properties compared to the most common ones at this position.

*Further evidence from genome scans* The association of this locus with milk composition traits has been replicated by genome-wide association studies [31–35] and a selective sweep has been reported in this region in several cattle breeds [36].

##### *DGAT1* (ENSBTAP00000037256:p.Ala323Lys)

Diaglyceride *O*-acyltransferase 1 (DGAT1) is the enzyme that catalyses the synthesis of triglycerides from diglycerides and acyl-coenzyme A. Grisart et al. [37] identified the missense mutation p.Ala323Lys in the bovine *DGAT1* gene associated with major effects on milk yield and composition.

*Location* The p.Ala323Lys variant is located on cattle chromosome 14 between positions 611,019 and 611,020 in ARS-UCD1.2. We confirmed the location by aligning the amino acid sequence from Fig. 3 of Grisart et al. [37] to the Ensembl peptide ENSBTAP00000037256 with Emboss Needle. However, the variant described by the original authors was a dinucleotide substitution, which Ensembl Variation represents as two SNVs (rs109326954 and rs109234250). When considered individually, the SNVs result in different amino acid substitutions (Ala > Thr and Ala > Glu) whereas when they occur together, they would cause Ala > Lys.

*Evidence for the causative gene DGAT1* is a functionally attractive candidate gene for milk composition traits, since it encodes an enzyme involved in triglyceride synthesis [37].

*Evidence for the causative variant* The original paper sequenced the *DGAT1* gene and detected one amino acid substitution, which was present on two haplotypes associated with high values for milk yield, protein content, fat content and fatty acid composition. In a later functional analysis [38], both *DGAT1* alleles were expressed in an insect cell line, and microsomes that carried the membrane-bound protein were used to measure the ability of the alleles to synthesise triglycerides. The Lys allele synthesised 1.5 times the amount relative to the Ala allele, which is consistent with the difference in milk composition.

*Further evidence from genome scans* Associations with milk traits at this locus have been replicated in genome-wide association studies [31, 33–35, 39–42].

##### *GHR* (ENSBTAP00000001758:p.Phe279Tyr and ENSBTAP00000001758:p.Asn528Thr)

Growth hormone plays critical roles in the control of lactation, mammary gland development, growth processes and fertility in cows [43, 44]. Growth hormone exerts its effects by interacting with a specific receptor (GHR) on the surface of target cells. In 2003, Blott et al. [45] identified two missense variants, ENSBTAP00000001758:p.Phe279Tyr and ENSBTAP00000001758:p.Asn528Thr, in the *growth hormone receptor* gene that displayed a strong association with milk yield and composition.

*Location* The two *GHR* variants are located on cattle chromosome 20 at positions 31,870,098 (p.Phe279Tyr) and 31,888,449 (p.Asn528Thr) in ARS-UCD1.2. They were mapped to the genome by Ensembl Variation. We confirmed their locations by comparing the amino acid sequence of Fig. 3 in [45] to the reverse complemented sequences flanking the variants in the reference genome.

*Evidence for the causative gene* Given its role in lactation, *GHR* is an attractive candidate gene [45].

*Evidence for the causative variant* Blott et al. [45] sequenced the coding sequence of the *GHR* gene and found two amino acid substitutions.

*Further evidence from genome scans* The association of this locus with milk traits has been replicated in genome-wide association studies [34, 35, 41] and there is evidence of positive selection at the locus [46].

##### *MSTN* (ENSBTAP00000015674.4:p.Asp273fs, ENSBTAP00000015674.4:p.Cys313Tyr, ENSBTAP00000015674.4:p.Gln204*, ﻿ENSBTAP00000015674:p.Gln204*, ENSBTAP00000015674.4:p.Phe140*)

Myostatin (MSTN) is a member of the transforming growth factor β superfamily of growth factors involved in the regulation of skeletal muscle [47]. The bovine *MSTN* gene causes the muscle growth ("double-muscled") phenotype in cattle. In 1997, several groups identified an 11-bp deletion in the bovine *MSTN* coding sequence, as the variant responsible for this phenotype in cattle [48–50]. In addition, another indel, two premature stop codons, and one missense variant were also proposed as causative variants [50, 51].

*Location* See Table 1 for locations of variants on the ARS-UCD1.2 cattle reference genome. We had to use different strategies to localise the variants:

- For p.Asp273fs, we searched for the allele with the indel and its flanking sequence of Fig. 3 of [48] and Fig. 1 of [49] in the reference genome with BLAT. We confirmed the mapping by manually entering the alleles into the web version of the Variant Effect Predictor. The deletion is listed in the Ensembl Variation database (with accession number *rs382669990*), but at position 6,283,673 and flagged because “None of the variant alleles match the reference allele”. This appears to be an alignment issue of the indel since the allele matched the reference if it was shifted by one bp.
- p.Gln204* was mapped to ARS-UCD1.2 by Ensembl Variation (with accession number *rs110344317*). We confirmed the location by comparing its flanking sequence with the sequence in Fig. 1 of [51].
- Both p.Glu226* and p.Cys313Tyr were mapped by searching for the sequences from Fig. 1 of [51] in the genome using BLAT. We confirmed the amino acid positions using the Ensembl REST API.
- p.Phe140* was mapped by extracting the flanking region before the indel from the coding sequence of *MNST* with GenBank accession *AF019761.1* (generated by [49]), and searching the genome for this flanking sequence with BLAT. (The sequence shown in Fig. 1 of [51] did not yield a hit in the genome sequence, probably because of its short length or because the reference genome carries a synonymous SNV a few bp before the indel) We confirmed the predicted premature stop codon with the Variant Effect Predictor.

*Evidence for the causative gene* Knockout of *MSTN* causes double muscling in multiple species, including cattle [52], pig [53–55], rabbit and goat [56], sheep [57], and mice [50, 58].

*Evidence for the causative variant* The indel and premature stop codons are predicted to cause a truncated protein, which is likely to cause loss of protein function. The missense variant lacks direct evidence of causality.

*Further evidence from genome scans* The locus has been associated with production traits [59] and calving ease [60] in genome-wide association studies. There is evidence of a signature of selection at this locus [61, 62].

##### *PRLR* (ENSBTAP00000069979:p.Ala461fs, ENSBTAP00000069979:p.Ser465* and ENSBTAP00000069979:p.Arg497*)

Autosomal dominant mutations in the *prolactin receptor* (*PRLR*) gene are associated with heat tolerance adaption and hair morphology phenotypes. These variants produce the thermotolerant shorter hair and lower follicle density ('slick') coats of the Senepol breed. The slick phenotype has previously been mapped by linkage mapping to a locus that overlaps with the *PRLR* gene [63]. Littlejohn et al. [64] proposed one causative variant in Senepol cattle. Porto-Neto et al. [65] sampled further breeds, and proposed two other causative variants.

*Location* See Table 1 for the location of the variants on ARS-UCD1.2. The reports [64] and [65] both contain genome coordinates on the UMD_3.1.1 cattle reference genome. To move them to ARS-UCD1.2, we used the mapping data provided by Ensembl Variation.

- For p.Ala461fs, we looked up the coordinate given [64] in the archived Ensembl release 94 (which used the UMD_3.1.1 reference genome), which allowed us to find the variants in Ensembl Variation. Then, we used the accession number to look for the variant in Ensembl Variant version 102. We confirmed the localization by aligning the amino acid sequence in Fig. 2 of [64] with the Ensembl peptide ENSBTAP00000069979.1 with Emboss Needle.
- p.Ser465* and p.Arg497* are not listed in Ensembl Variation. We used UCSC LiftOver to map the coordinates between genome versions, and the Ensembl REST API to check the position of the codon (map/translation endpoint).

*Evidence for the causative gene Prolactin* was an attractive candidate gene because of the isolation of a putative loss-of-function variant by [64] that associated with the ‘hairy’ phenotype in cattle, and because prolactin signalling is involved in hair growth (reviewed by [66, 67]).

*Evidence for the causative variant* Littlejohn et al. [64] sequenced the *PRLR* gene in purebred Senepol cattle and identified a single base deletion in exon 10 that causes a frameshift and introduces a premature stop codon. This variant co-segregated with the slick phenotype in 82 crossbred cattle [64]. Porto-Neto et al. [65] genotyped this variant in slick-coat cattle from other breeds and found individuals with a genotype that was discordant with the trait. They sequenced these cattle, performed a genome-wide association study and identified two more putative *PRLR* loss-of-function variants (p.Ser456* and p.Arg497*) that may explain the slick coats of these cattle.

*Further evidence from genome scans* The slick phenotype has been associated with the same region in genome-wide association studies [68] and there is evidence of a signature of selection at the locus [46].

#### Pig

##### *IGF2* (*NC_010444.4:g.1483817T* > *C*)

*Insulin-like growth factor 2* (*IGF2*) is a paternally expressed imprinted gene that promotes growth and plays an important role in proliferation, differentiation and apoptosis of cells in different tissues [69]. A locus for muscle mass in pigs that overlaps with *IGF2* was mapped independently in experimental intercrosses [70, 71]. Later, IGF2 was proposed as a physiological regulator of preadipocyte growth, metabolism and body fat composition in humans [72, 73]. In 2003, Van Leare et al. [74] described *g.1483817T* > *C* as the potential causative variant.

*Location* The *g.1483817T* > *C* variant is located on pig chromosome 2 at position 1,483,817 in Sscrofa11.1. As this variant is noncoding, we could not use predicted effects on protein sequence to localize it. Instead, we searched for the flanking DNA sequence from Fig. 1 of [74] in Sscrofa11.1 using BLAT. A matching sequence was found in the third intron of the Ensembl transcript *ENSSSCT00000050274.2*, which is consistent with the description in the original paper. However, with respect to other Ensembl *IGF2* transcripts, this might be also be the fourth, fifth or sixth intron. The original paper [74], which predates the pig reference genome and gene annotation, refers to the variant as “intron3-3072”, and a pairwise Emboss Water alignment between the flanking sequence and intron 3 of *ENSSSCT00000050274.2* places the variant at position 3071. In the original paper, the SNV is described as G > A; this corresponds to T > C on the reference genome, since the reference pig carries allele *A* and *IGF2* is transcribed in reverse orientation with respect to the reference genome.

*Evidence for the causative gene IGF2* is a functionally attractive candidate given its known function in myogenesis, growth and development. The locus also showed evidence of genomic imprinting with a paternal expression pattern that is consistent with the maternal imprinting at *IGF2* [70, 71]. Gene expression analysis by Northern blot showed an expression difference between the two alleles in skeletal muscle [74].

*Evidence for the causative variant* The variant occurs in a conserved noncoding sequence. The original paper [74] used an alignment of eight vertebrate sequences. It overlaps a constrained region detected by GERP in Ensembl’s 95-way vertebrate alignment. An electrophoretic shift mobility assay and a reporter expression assay, both performed in a mouse cell line, showed differences in protein-binding and expression between the two alleles [74]. A later study identified the protein that binds with this noncoding sequence by showing that the single nucleotide substitution *g.1483817T* > *C* abrogates the binding site for ZBED6, a nuclear factor which represses *IGF2* transcription [75].

*Further evidence from genome scans* Genome-wide association studies reported growth-related associations on a distal region of pig chromosome 2 [76, 77]. However, the original locus showed evidence of genomic imprinting, and one of these associations did not. On the previous version of the pig reference genome, Sscrofa10.2, the *IGF2* gene was not anchored on chromosome 2 but on an unassigned sequence. This might have impeded replication by genome-wide association. The genetic association with *IGF2* expression in skeletal muscle at this locus has been replicated by genome-wide expression QTL mapping [78].

##### *MC4R* (ENSSSCP00000074588.1:p.Asp298Asn)

Melanocortin-4-receptor (MC4R), a G-protein-coupled receptor expressed in the brain, has a fundamental role in regulating food intake and energy expenditure [79]. Leptin acts on the central nervous system to cause a reduction in food intake and body weight. MC4R receptor signalling mediates the effect of leptin on food intake and energy homeostasis and has been implicated in the regulation of feeding behaviour and body weight in humans and mice, with agonists of MC4R reducing food intake [80] and targeted mutation of *MC4R* causing obesity [81].

In 2000, Kim et al. identified a missense variant (p.Asp298Asn) in the pig *MC4R* gene, which is associated with growth and fatness traits [82]. This study is a rare example of a successful candidate gene study that, by picking a candidate gene a priori, was able to identify both a major locus for a complex trait and a potential causative variant that shows molecular evidence of function. Notably, the original study used a relatively large number of pigs (sample sizes of 1740, 1194 and 231 for different traits) from six lines of different breeds, thus providing both potential for good power and replication between different populations.

*Location* The variant is located on pig chromosome 1 at position 160,773,437 in Sscrofa11.1. It was mapped to the pig genome by Ensembl Variation. We confirmed the mapping by aligning the amino acid sequence in Fig. 1 of [82] to the Ensembl peptide ENSSSCP00000074588.1 with Emboss Water.

*Evidence for the causative gene MC4R* was selected a priori as a candidate gene based on its known function in body weight and obesity in other species.

*Evidence for the causative variant* Kim et al. [82] sequenced the pig *M4CR* gene and identified one missense mutation, which was significantly associated with backfat, growth-rate and feed intake in commercial pig lines. A follow up-study carried out a functional analysis of the *MC4R* variant by expressing both alleles in a human cell line [83]. MC4R binds to the G-protein as a cAMP-dependent pathway activator. When stimulated with its ligand (an analogue of melanocyte stimulating hormone), the alleles showed a difference in cAMP production, but no difference in ligand binding. This suggests that the Asn allele causes loss of the normal receptor function and a decrease in melanocortin signalling.

*Further evidence from genome scans* The locus was subsequently found in linkage mapping [84–87] and genome-wide association studies [88, 89] and there is evidence of a signature of selection in domestic pigs [90].

##### *MSTN* (ENSSSCP00000017001:p.Glu274*)

In the pig, in addition to double muscling, the *MSTN* gene has been associated with leg weakness. Matika et al. [91] mapped a leg weakness syndrome that causes piglet mortality, in a commercial pig line. Segregation analysis suggested a major recessive locus, and homozygosity mapping was performed in case animals and related control animals and an 8-Mb candidate region was identified. Sequencing identified a SNV that causes a premature stop codon in *MSTN*. Matika et al. [91] also estimated the associations of this variant with several production traits that have been under selection in this population. The results suggest that balancing selection can explain the high frequency of the damaging variant (22%).

*Location* The p.Glu274* variant is located on pig chromosome 15 at position 9,4623,834 in Sscrofa11.1, as reported by [91].

*Evidence for the causative gene* While knockout of the *MSTN* gene results in increased muscle growth in pigs, experimental knockout animals have also shown severe leg weakness and early mortality, as reported in one paper [92], but not in others [55, 93, 94].

*Evidence for the causative variant* The SNV introduces a premature stop codon, which is likely to cause loss-of-protein function. A histological comparison of homozygous and heterozygous animals suggested hypertrophy of muscle fibre, which is consistent with loss of *MSTN* function.

##### *NR6A1* (ENSSSCP00000005986:p.Leu192Pro)

In pigs, the number of vertebrae varies and is associated with meat productivity. Wild boars, which are the ancestors of domestic pigs, have 19 vertebrae. European commercial pig breeds have 21 to 23 vertebrae, probably as the result of selective breeding for increased body size. Two linkage mapping studies in different intercrosses detected a locus for vertebrate number on pig chromosome 1 [95, 96]. After fine-mapping, a missense variant in the *nuclear receptor subfamily 6 group A member 1* (*NR6A1*) gene was proposed as the causative variant [97].

*Location* The p.Leu192Pro variant is located on pig chromosome 1 at position 265,347,265 in Sscrofa11.1. The variant was mapped to the genome by Ensembl Variation. We confirmed the mapping by pairwise alignment of the amino acid sequence from Fig. 3 in [97] to the Ensembl peptide ENSSSCP00000005986.3 with Emboss Needle.

*Evidence for the causative gene NR6A1* is an attractive candidate gene because of its role in embryonic development. It is expressed widely in early mouse embryos and later in the developing nervous system [97, 98]. Mutant embryos display serious defects in somitogenesis with a maximum of 13 (instead of 25) somites [99].

*Evidence for the causative variant* Mikawa et al. [97] sequenced the coding regions of two genes in the regions and found one missense variant in *NRGA1* co-segregating with the locus. *NR6A1* is a transcriptional repressor, which recruits various corepressor complexes to repress and silence gene transcription. The missense variant occurs in the hinge domain, which is essential for the interaction of NR6A1 with two corepressors, i.e. the nuclear receptor corepressor 1 (NCoR1) [100] and the nuclear receptor associated protein 80 (RAP80) [101]. A two-hybrid assay suggests that p.Leu192Pro is a gain-of-function mutation in the hinge domain, as it increases the interaction between NR6A1 and NCoR1 and the interaction between NR6A1 and RAP80.

*Further evidence from genome scans* Association with vertebrae number has been replicated in a genome-wide association study [102] and there is evidence of a signature of selection in domestic pigs [90].

##### *PHKG1* (NC_010445.4:g.16830320C>A)

The *PHKG1* gene encodes a catalytic subunit of the phosphorylase kinase (PhK), which functions in the cascade activation of glycogen breakdown. Ma et al. [103] identified a splicing mutation in *PHKG1*, which they propose as a causative variant for glycogen content and meat quality in pig skeletal muscle.

*Location* The variant is located on pig chromosome 3 at position 16,830,320 in Sscrofa11.1. Ma et al. [103] refer to *g.16830320C* > *A* as *g.8283C* > *A*, and deposited the coding sequence in GenBank under accession *KJ481910.1*. We aligned the sequence flanking this position in the coding sequence to the pig reference genome using BLAT. The variant was located 5 bp before the start of exon 10 of *PHGK1* (Ensembl Transcript *ENSSSCT00000008491.4*), which is consistent with the original article. Ma et al. [103] uses the accession *ss131031160*, which is found neither in Ensembl Variation nor in EVA.

*Evidence for the causative gene PHKG1* is an attractive candidate gene because of its known role in glycogen breakdown and an association between genotype and *PHKG1* expression. Ma et al. [103] performed expression QTL mapping using muscle transcriptome data from 497 pigs and detected an association with *PHKG1* expression at the locus. This is consistent with a variant that affects *PHKG1* expression which in turn affects glycogen content. They also measured phosphorylase kinase enzyme activity in muscle samples from genotyped pigs and found a difference in enzyme activity between alleles at the locus.

*Evidence for the causative variant* Sequencing of the *PHKG1* cDNA detected a 32-bp frameshift deletion in exon 10 which causes a premature stop codon. Ma et al. [103] did not find this deletion in the genomic DNA, but they identified the *g.16830320C* > *A* variant and hypothesised that it might be a splice variant. Splicing assays in two human cell lines (HeLa and 293 T) showed that *g.16830320C* > *A* is responsible for the aberrant splicing of 32 nucleotides observed in exon 10 of *PHKG1*. The variant also reduced *PHKG1* mRNA expression, which is consistent with the local eQTL study, where the alleles differed in their *PHKG1* expression level. Ma et al. suggested that the truncated *PHKG1*, expressed at 56% of the expression level of the normally spliced allele, is most likely degraded by nonsense-mediated decay [103].

##### *PRKAG3* (ENSSSCP00000017163:p.Arg250Gln)

Glycogen storage diseases are a group of inherited disorders that are characterised by excess glycogen storage and are primarily caused by abnormalities in an enzyme responsible for releasing glucose from glycogen. Pigs affected by a glycogen storage disease produce inferior meat with a lower pH (so-called "acid meat") and a lower processing yield due to post-mortem degradation of excess glycogen (reviewed in [103, 104]. The variant responsible for this phenotype is also known as the “rendement napole” or “RN^–^ gene”. A mutation in the *protein kinase AMP-activated non-catalytic subunit gamma 3* gene (*PRKAG3*) has been proposed as the causative variant (p.Arg250Gln) for abnormal glycogen content in pig skeletal muscle [104]. The *PRKAG3* gene encodes a regulatory subunit of the 5' adenosine monophosphate-activated protein kinase (AMPK).

*Location* The p.Arg250Gln variant is located on pig chromosome 15 at position 120,863,533 in Sscrofa11.1. To localise this variant, we aligned the amino acid sequence from Fig. 1 in [104] to the Ensembl peptide ENSSSCP00000017163 with Emboss Needle. In the original paper, the variant was reported as Arg200Gln on a shorter protein sequence; our pairwise alignment placed it at Arg250Gln on the current sequence. We used the Ensembl REST API endpoint GET map/translation/:id/:region to map the corresponding codon to the genome coordinates 15:120,863,532–120,863,534. Table 3 in [104] shows that the SNV is located at the middle base of the codon.

*Evidence for the causative gene* The *PRKAG3* gene is a functionally attractive candidate because the γ3-subunit of AMPK plays a key role in regulating carbohydrate and fat metabolism in mammalian skeletal muscle cells, and is primarily expressed in white skeletal muscle fibres [105]. It has been reported that a loss-of-function mutation in a yeast homolog produces defective glycogen storage [106, 107]. Proteomic analysis suggests that the glycogen accumulation is caused by increased glycogen synthesis, which is consistent with a constitutively active AMPK [108].

*Evidence for the causative variant* In the original study, p.Arg250Gln was the only amino acid substitution detected that was associated with the trait [104].

*Further evidence from genome scans *Association of this locus with meat quality traits has been replicated by genome-wide association studies [109–112].

##### *RYR1* (NP_001001534.1:p.Arg651Cys)

Malignant hyperthermia is an inherited, potentially lethal pharmacogenetic disorder in which sustained muscle contracture, with attendant hypercatabolic reactions and elevated body temperature, are triggered by commonly used inhalation anaesthetics and skeletal muscle relaxants [113]. In pigs, malignant hyperthermia is a serious economic problem as it leads to sudden, stress-induced deaths and to pale soft, exudate meat. A single mutation in the skeletal muscle Ca2+ -release channel gene, *ryanodine receptor 1* (*RYR1*), has been reported as causative of malignant hyperthermia [114].

*Location* The p.Arg651Cys variant is located on pig chromosome 6 at position 47,357,966 in Sscrofa11.1. To localise this variant, we searched the Sscrofa11.1 database for the DNA sequence in Fig. 1 of [114] with BLAT. This yielded a unique hit in the chromosomal region where the *RYR1* gene is mapped in the NCBI gene annotation. We verified the mapping by searching for the amino-acid sequence in Fig. 1 of [114] in the RefSeq protein sequence *NP_001001534.1*. The *RYR1* gene is missing from the current Ensembl gene annotation (version 102), but included in NCBI/RefSeq.

*Evidence for the causative gene RYR1* is a functionally attractive candidate gene in pig because it is associated with a similar malignant hyperthermia syndrome in humans [115], and it is involved in the regulation of calcium release in the skeletal muscle. A functional study of the sarcoplasmic reticulum vesicles obtained from pigs that were homozygous for the opposite allele at this locus showed that the allele associated with malignant hyperthermia resulted in higher ryanodine affinity and higher calcium-induced calcium release activity [116]. This is consistent with a difference in RYR1 function between alleles.

*Evidence for the causative variant* Fuji et al. [114] found that the SNV in the *RYR1* gene was correlated with susceptibility to malignant hyperthermia in five pig breeds. Otsu et al. [117] showed that halothane resistance and the SNV co-segregated in 182 pigs from six breeds.

##### *SYNGR2* (ENSSSCP00000041695:p.Cys63Arg)

Porcine circovirus 2 (PCV2) is a DNA virus responsible for a group of systemic disorders that are collectively known as PCV2 associated diseases. A genome-wide association study of viral load in crossbred pigs challenged with PCV2 identified two loci [118]. One of these regions was fine-mapped to the *synaptogyrin-2* (*SYNGR2*) gene, and a missense variant, R63C, was detected within this gene. *SYNGR2* is a non-neural member of the synaptogyrin gene family, which contains genes that are expressed in the membrane of synaptic vesicles [119].

*Location* The p.Cys63Arg variant is located on chromosome 12 at position 3,797,515 in Sscrofa11.1. In [118], although the amino acid position was specified, there was no accession number for the *SYNGR2* sequence used. We mapped the codon to the genome coordinates 12:3,797,513–3,797,515 in Sscrofa11.1 with the Ensembl REST API. We compared the amino acid sequence in Fig. 4 of [118] with the Ensembl peptide ENSSSCP00000041695.1 to confirm the amino acid position. In order to identify which nucleotide in the codon is the SNV, we compared codon 63 of the *SYNGR2* gene in the Landrace, Large White, and Meishan genomes annotated by Ensembl (all reported to carry the Arg allele) with that of the reference genome (carrying the Cys allele) and found that the former all carry CAG, while the reference genome carries TAG. This suggests that the SNV is in the first position of the codon (in reverse orientation relative to the reference genome).

*Evidence for the causative gene* Based on RNA sequencing data, *SYNGR2* was shown to be expressed in the peripheral blood from pigs subject to PCV2 [118]. Previously, Sun et al. [120] demonstrated that SYNGR2 has a role in the replication of a tick-borne human RNA virus. In vitro silencing of *SYNGR2* expression in pig cells, using siRNA and CRISPR-Cas9 editing, caused a significant reduction in PVC2 titer, which confirmed the role of SYNGR2 in viral replication [118].

*Evidence for the causative variant* Variant calling from RNA sequencing data found one missense variant, p.Cys63Arg, in a conserved domain of *SYNGR2*. Fine-mapping in pigs with high and low viral loads (n = 268) revealed that the associations with viral load were strongest for this missense variant and an indel close to the *BIRC5* gene. Since these two variants were in high linkage disequilibrium, it was difficult to distinguish them by fine-mapping. Walker et al. [118] applied CRISPR-Cas9 editing to remove the region containing the p.Cys63Arg variant, which led to a frameshift and the production of an altered protein. This supports *SYNGR2* as the causative gene, and thus indirectly the p.Cys63Arg variant. Moreover, the *BIRC5* gene was not differentially expressed, which is indirect evidence against the *BIRC5*-adjacent indel as a gene regulatory variant.

##### *VRTN* (g.97614602A > C and g.97615879_97615880ins)

In addition to the *NR6A1* locus, another major locus has been shown to affect vertebrae number in pigs due to variants in the *VRTN* gene. Mikawa et al. [121] fine-mapped this locus in multiple intercrosses of European and Asian pig breeds and a commercial cross, and identified the *VRTN* gene.

*Location* Fan et al. [102] report the position of the proposed causative variants on the *VRTN* sequence with accession *AB554652.1* (which was generated by [121]). We extracted the flanking region upstream of these variants and searched the Sscrofa11.1 reference genome with BLAT. We found positions that agree with those in [122], also based on *Sscrofa11.1*. We aligned a 2000-bp region flanking this position from the *AB554652.1* sequence to the corresponding region from the Sscrofa11.1 reference genome with Emboss Needle, and confirmed that *AB554652.1* contains a 291-bp insertion that is absent in the reference genome.

*Evidence for the causative gene* Mikawa et al. [121] fine-mapped the candidate region to a 41-kb region that overlaps *VRTN*, and Fan et al. [102] fine-mapped it to 100-kb region that overlaps with both *VRTN* and a neighbouring gene. Allele-specific expression in heterozygous pig embryos by reverse transcription PCR, cloning and sequencing revealed a difference in *VRTN* expression between alleles [121]. Reporter assays in mouse and pig embryos showed that *VRTN* was expressed along the anterio-posterior axis, while *VRTN* knockout mice showed defects in vertebrae development [123].

*Evidence for the causative variants* Initially, sequencing of the 41-kb candidate region identified nine variants that were concordant with the locus, which were later refined to four candidate variants [102, 121]. Dual reporter assays in a human cell line showed that the two *g.97614602A* > *C* and *g.97615879_97615880ins* variants drove reporter expression, additively and with approximately equal effects, whereas the other two candidate variants did not [123].

*Further evidence from genome scans* After the initial multiple linkage mapping studies, the locus was also detected by genome-wide association for vertebrae and teat number in pigs [102, 122].

##### Methods used to localize variants

As the above sections show, we used several strategies to search for and verify the location of variants in the genomes. Most of the time, we could rely on the Ensembl Variation database and the consequences of the variants predicted with VEP to map them to contemporary reference genomes and gene annotation. When the variants were not available in the Ensembl Variation database (or EVA), either we used the UCSC LiftOver tool to move variants between reference genomes (when there was a genome coordinate but on an older version of the reference genome) or we aligned the nucleotide sequences to the reference genome with BLAT.

Frequently, the original publications did not contain genomic coordinates because the results were generated before a reference genome was available, or accession numbers for the cDNA and amino acid sequences used. This means that local coordinate systems, such as those that indicated the position with respect to a start site identified only by a gene name or exon number, were of limited use. The risk of such descriptions is to have an ambiguous localisation if the gene annotation changed to include differently spliced transcripts. However, today there is little reason not to report variants with reference genome coordinates.

Perhaps unexpectedly, DNA and protein sequences reported in figures of the original papers turned out to be useful to be able to align alleles to reference genomes and confirm their localization. The fact that most protein-coding variants could be localized based on amino acid positions suggests that while the noncoding sequence around genes may have changed with updated genome assemblies, the predicted gene structures and amino acid sequences used in the original papers correspond well with contemporary gene annotation. The exception is the *PRKAG3* p.Arg250Gln variant, which was found to be shifted by 50 amino acids compared to the sequence used in [104].

In some cases, such as the *DGAT1* multinucleotide variant *NC_037341.1:g.611019_611020delinsAA* and the *MSTN* deletion *NC_037329.1:g.6281243_6281249delinsAAGCATACAA*, variants were more complex than a simple SNV, which complicated the information found in variant databases. For example, the functional consequence of the multinucleotide variant in *DGAT1* differed from that of the two variants considered as individual SNVs. Also, the position of the indel candidate for *MSTN* appeared to be offset by one bp compared to its actual position in the genome. These are relatively simple non-SNV variants compared to large-scale structural variants such as tandem repeats or inversions. This suggests that structural variant data will be a real challenge for current variant databases and annotation methods.

##### Suggestions for reporting new potential causative variants

Based on these observations, we make the following recommendations for reporting the position of new potential causative variants:

- *Use referenced coordinate systems* When referring to a change in a genomic DNA sequence, the coordinates should be based on a publicly available reference genome, and the version of the reference genome used should be stated. This is going to become more and more important, as more alternative genomes for different breeds are published. When referring to a change in a protein coding sequence, the accession number of the specific isoform used (e.g., Ensembl Transcript/Peptide IDs or RefSeq accession numbers) should be included.
- *Show local sequence context* It is useful to continue to provide DNA sequences and amino acid sequence alignments in the figures of a publication (it would be even better to also include them in a reusable file format in the supplementary data, so that they do not need to be extracted from images). Such figures can be surprisingly helpful for checking mapping positions to reference genomes.
- *Submit variants to repositories* If possible given the size of datasets and potential restrictions, the variant datasets, including the proposed causative variants, should be submitted to a variant repository (such as EVA). In this way, the variants will be searchable, and kept mapped to up-to-date reference genomes, as well as potentially variant annotation databases. If submission to a dedicated variant repository is not possible or dataset size is prohibitive, regional datasets that cover the proposed causative genes and some of their flanking regions could be submitted to general data repositories. Reference genome coordinates and bioinformatic file formats have made some aspects of reporting easier than previously, but dataset size and interoperability will remain a challenge.

Naming potential causative variants relies on variant annotations, i.e. on the prediction of their function. Often, the annotations of variants indicate relatively simple consequences, but they may also include molecular evolutionary statistics such as conservation scores, and in the future, more sophisticated predictions based on the output of statistical and machine learning models. One possibility for reporting this kind of information in a standardised way would be to put them in the INFO field of the variant call format [124], as supported by several software packages that integrate the functional consequences of variants.

Including the sequences around the variant alleles should be particularly useful for non-SNV variants, such as insertions and deletions, or even larger structural variants for which the file formats used for encoding purposes are less standardised. The same indel can be encoded in different ways in variant call files [125], and thus including the sequences that flank them could provide extra insurance against potential misalignment of indels.

With the increasing number of livestock genome assemblies and versions, researchers might adopt pangenome references that catalogue structural diversity within a species (e.g. [126, 127]) and representations of graph genomes that store such pan-genome information in a single data structure [128]. Graph genomes allow bioinformatic methods (e.g. sequence alignment and variant calling) to deal with genomic diversity in a principled way; but on the downside, they remove the simple linear coordinate systems of traditional reference genomes. It will become even more important to document what version of the (pan-)genome is used, and to have tools to go from one genome assembly version to another.

##### Strength of the evidence that supports the proposed causative variants

Taken together, there is usually good evidence for the association at the locus level, some evidence for a specific causative gene at eight of the loci, and some experimental evidence for a specific causative variant at six of the loci. Often the detected loci have been replicated by genome-wide association studies or linkage scans, which reinforces the confidence in the genetic effects associated with these loci.

Causative genes are often supported by functional evidence reported for similar traits or biological processes in other species, and in some cases, by direct evidence from knockout experiments (such as for *MSTN* or *SYNGR2*) that show that the gene affects relevant physiological processes. However, even strong confirmation such as that provided by knockout experiments does not necessarily demonstrate genetic causation. For example, consider the *FTO*/*IRX3* locus in humans. The observed association between an intronic variant in *FTO* and obesity [129, 130] is explained by the presence of an enhancer within this intron that interacts with the neighbouring *IRX3* gene and alters its expression with an impact on obesity. Thus, this result suggests that *FTO* itself is not the causative gene at this locus in humans, although previous studies based on the knockout or overexpression of *FTO* in mice [131, 132] showed that it affected body mass. Hopefully, such situations of misleading neighbouring functional genes are rare.

The evidence that supports the specific causative variants examined in the current paper was generally weak. For six of the 14 loci, there are follow-up experiments that test the functions of the variants experimentally, by testing its effect on some aspect of protein function or gene expression. Some examples are: (1) the reporter and protein-binding assays that suggested that the variant *NC_010444.4:g.1483817T* > *C* in the *IGF2* gene affects transcription-factor binding and *IGF2* expression [74, 75]; (2) the splicing assay showing that the splice site-adjacent variant *NC_010445.4:g.16830320C* > *A* detected in the *PHKG1* gene affects splicing [103]; and (3) functional studies of the *MC4R* variant by in vitro expression suggesting that the alleles differ in their ligand affinity and second messenger production [83]. In all these cases, the evidence for the molecular function combined with that for the analysed causative gene constitutes strong support for causality, even in the absence of a direct test of the effect of the variant on the traits at the organism level. In other cases (such as *MSTN* and *PRLR*), the variants are frameshift variants, which are a priori likely to affect gene function. However, in cases where the evidence consists only of the identification of a missense variant, the causative variant could be another variant in close linkage, especially when the sequencing data is limited to coding regions or to selected candidate genes.

In some of these cases, multiple causative variants are likely. For example, for the *VRTN* gene, the functional evidence implicates two noncoding causative variants, but there is also evidence of genetic heterogeneity at the locus between breeds, which might be due to other causative variants in linkage disequilibrium [122]. Multiple potential loss-of-function variants have been observed both in cattle, i.e. in the case of *MSTN* for double muscling and *PRLR* for the slick phenotype. In the case of the *PRKAG3* gene, Uimari et al. [133] detected a locus that overlapped with this gene in Finnish Yorkshire pigs that cannot be caused by the previously proposed p.Arg250Gln variant because it was fixed in the population, i.e., all the Finnish Yorkshire pigs carried the same allele. Instead, sequencing of the gene identified multiple coding and noncoding variants in strong linkage disequilibrium [134] with each other.

Several papers (e.g. [37, 45, 104]) used multiple sequence alignments of homologous genes from other species to determine whether the nucleotide substitution in question is conserved or not, but it is unclear what weight should be given to these informal evolutionary analyses, as they used different, usually small, selected homologous sequences, but did not apply an evolutionary model beyond multiple sequence alignment. For example, Grisart et al. [37] aligned homologous sequences of the *DGAT1* gene from eight species, and concluded that the position of their p.Ala323Lys substitution was “conserved” because it was shared by seven of them. An example of the use of a formal evolutionary model is in [102], where the authors analysed the homology with human open chromatin and scores from the GERP [135] method to assess conservation of two potential gene-regulatory variants. More recent sophisticated variant effect prediction methods include both conservation and other genomic information (see next section), and might provide more reliable information.

In this paper, most of the causative variants that we have examined are fairly old, maybe because the focus of research has changed from gene mapping to genomic selection and has increased standards of evidence, or because the low hanging fruits of large effect variants have already been picked. We should also add that our selection of the literature is a convenience sample and not a systematic review. For example, in Walker et al. [118] the evidence that supports their *SYNGR2* missense variant may be as good as that of many of the older papers but the authors seem to be much more cautious about advertising it as a causative variant, probably because they are aware of the genomic complexity and of the multiple ways they might be wrong.

##### Developments that will likely improve the identification of causative variants

There are three ongoing developments in genomics that have the potential to transform the identification of causative variants for complex traits: more comprehensive functional genomic datasets for prioritising noncoding variants, more sophisticated computational predictors of the function of variants, and high-throughput experimental assays of variant function. However, in all cases, there are challenges for livestock genomics.

Noncoding gene-regulatory causative variants present specific challenges for the identification of causative variants. In contrast to protein-coding variants, which are more amendable to functional classification from sequence data and gene annotation, noncoding variants are more difficult to classify. While there are some regularities in gene-regulatory sequences (e.g. core promoter features such as the TATA box and transcription factor binding sites that can be summarised as position-specific weight matrices), these patterns are too variable for searches of whole-genome motifs to have acceptable statistical properties [136]. Thus, the field regarding the identification of non-coding variants has turned to gathering functional genomic data. While expression quantitative trait data is limited by linkage disequilibrium in the same way as genetic mapping, these data can be compared to genetic mapping results of traits for the detection of putative causative genes, and be enrichment-tested to identify putatively causative tissues and cell types for complex traits (e.g. [11] reported enrichment analyses of liver-expressed genes for ketosis in cattle, and of mammary gland-expressed genes for milk yield). Furthermore, chromatin sequencing data can be used to identify active gene-regulatory elements and infer gene-regulatory relationships that can be used for fine-mapping of noncoding variants (as in humans [137]). One challenge is that such methods require a wide and comprehensive coverage of tissues and cell types, which is significantly easier to obtain in humans and model organisms for which tissue and cell lines collections and protocols for induced pluripotent stem cells are more developed.

Another improvement for the identification of causative variants is the development of sophisticated variant effect prediction methods, both for coding and noncoding variants. Examples of recent approaches include MutPred2 [138], which predicts the effects of protein-coding variants based on bioinformatic models of protein sequence, sequence conservation, and a training set of monogenic disease variants from humans; FAETH [12], which prioritises variants in cattle based on the variance of complex traits explained by variants carrying similar annotations (from chromatin sequencing, expression and metabolite QTL mapping, variant annotation and sequence conservation); and pCADD [139], which is trained to predict deleterious variants in the pig genome by distinguishing simulated de novo mutations from variants that have been observed in sequence data. One major challenge is that these methods cannot be trained on known causative variants for complex traits, because there are so few; instead, the models solve related problems (detecting monogenic disease variants in humans, or predicting per-SNV heritability of molecular traits, or detect deleterious mutations). To use these methods for the identification of causative variants for complex traits, we need to assume that these methods are also accurate for this different problem. Recent evidence from applying pCADD to known causative variants in the pig is promising: Derks et al. [140] performed a genome-wide association study in purebred pigs from a commercial breeding program, extracted sequence variants in linkage disequilibrium with the most significant SNV, and ranked them by pCADD scores. In the case of *MC4R*, the putatively causative missense p.Asp298Asn variant was the top ranked variant at the locus. In the case of *PRKAG3,* the candidate missense variant first identified in Finnish pigs [134] was the top ranked variant at the locus. In the case of *VRTN*, the putatively causative promoter variant *NC_010449.5:g.97614602A* > *C* was the second highest ranking variant in one of the populations examined, and the fourth highest ranking in another. Similarly, the original pCADD paper [139] found the putative causative variant p.Leu192Pro in *NR6A1* to be in the top 90% of variants in the region.

Finally, developments in genome editing technologies and CRISPR-Cas9 screens now provide researchers with a host of strategies to modify candidate causative variants, in physiologically relevant contexts, either in vitro in cell culture or in vivo in animal models. These methods make it feasible to investigate the functions of variants, the target genes and most importantly, their role in the determination of the original phenotype. CRISPR-Cas9 and other gene editing technologies make it possible to both knockout genes (with non-homologous end joining) and substitute alleles (with homology-directed repair), but also to manipulate gene expression in cells without editing the DNA sequence. Most recently, work has focused on such assays for variants within non-coding regions of the genome. CRISPR-based assays can use guide RNAs to bind specific regions of the genome and either activate (CRISPRa) or interfere (CRISPRi) with the transcription of genes or enhancers [141–143]. Advances in single-cell RNA-seq and CRISPRi/a have further facilitated methodologies that evaluate enhancer effects on genes in single cells [144]. In livestock, the primary challenge for the application of CRISPR-Cas9 screening technology for genotype–phenotype analyses remains the paucity of available trait-relevant in vitro cell systems, the tissue specificity and development of which currently lag far behind that for human and model organisms.

There are several examples of gene editing applied to test causative genes or variants in livestock, in vitro and in vivo. Much of this work was performed not for the purpose of demonstrating the causality of variants, but to develop proposed applications of genome editing in animal breeding; see Tait-Burkard et al. [145] for a recent review of the topic. CRISPR-Cas9 disruption of the *SYNGR2* gene with CRISPR-Cas9 in a pig cell line to test its function in porcine circovirus 2 infection has been discussed above [118]. Similarly, disruption of the whole *CD163* gene [146], or the removal of only one of its exons [147], has demonstrated the role of *CD163* in porcine reproductive and respiratory syndrome virus infection. In vitro embryo production and CRISPR-Cas9 disruption have been used to demonstrate that loss-of-function of the *IFT80* gene (which is the proposed causative gene for a recessive lethal haplotype in Holstein cattle) is embryonic lethal [148]. As discussed above, knockout experiments of the *MSTN* gene have shown that it results in double-muscling phenotypes in several mammals, including cattle [52] (using zinc-finger nucleases) and pigs [54, 93, 94]. The generation of gene-edited calves that carry the *polled* allele (using the TALEN technology) has confirmed that it causes hornlessness [149]. The generation of gene-edited chickens with the CRISPR-Cas9 system has demonstrated that the *PMEL17 dominant white * allele causes white pigmentation of the feathers and the *KRT75 frizzled* allele causes brittle frizzled feathers [150]. Niu et al. [151] used CRISPR-genome editing to inactivate all of the porcine endogenous retroviruses (PERV) in a porcine primary cell line and generated PERV-inactivated pigs via somatic cell nuclear transfer.

## Conclusions

Causative variant identification remains a difficult problem. At six of the 14 loci reviewed in this paper, there is some experimental evidence supporting the function of a specific causative variant. In others, there is usually good evidence for association at the level of the locus, and sometimes for a particular gene. There are three ongoing developments—more comprehensive functional genomic datasets, more sophisticated computational predictors of the function of variants, and high-throughput experimental assays of variant function—that we believe will lead to an increasing rate of causative variant identification. However, in all three cases, there are challenges for livestock genomics, namely a smaller amount of functional genomic data than for humans and model organisms and a lack of cell biology resources such as cell lines. Localizing variants from the literature in contemporary reference genomes required several different kinds of bioinformatic strategies and searches. We recommend that authors proposing new causative variants use referenced coordinate systems, show local sequence context, and submit variants to repositories to make this process easier.

## Data Availability

Not applicable.
